# Modular AUV System with Integrated Real-Time Water Quality Analysis

**DOI:** 10.3390/s18061837

**Published:** 2018-06-05

**Authors:** Mike Eichhorn, Christoph Ament, Marco Jacobi, Torsten Pfuetzenreuter, Divas Karimanzira, Kornelia Bley, Michael Boer, Henning Wehde

**Affiliations:** 1 Institute for Automation and Systems Engineering, Technische Universität Ilmenau, Helmholtzplatz 5, 98693 Ilmenau, Germany; christoph.ament@informatik.uni-augsburg.de; 2 Fraunhofer IOSB-AST, Am Vogelherd 50, 98693 Ilmenau, Germany; mja@atlasmaridan.com (M.J.); torsten.pfuetzenreuter@iosb-ast.fraunhofer.de (T.P.); divas.karimanzira@iosb-ast.fraunhofer.de (D.K.); 3 -4H- JENA Engineering GmbH, Mühlenstraße 126, 07745 Jena, Germany; kornelia.bley@web.de (K.B.); boer@4h-jena.de (M.B.); 4 Institute of Marine Research, Nordnesgaten 50, 5005 Bergen, Norway; henningw@imr.no

**Keywords:** autonomous underwater vehicle, water quality monitoring, in situ-detection of nitrates

## Abstract

This paper describes the concept, the technical implementation and the practical application of a miniaturized sensor system integrated into an autonomous underwater vehicle (AUV) for real-time acquisition of water quality parameters. The main application field of the presented system is the analysis of the discharge of nitrates into Norwegian fjords near aqua farms. The presented system was developed within the research project SALMON (Sea Water Quality Monitoring and Management) over a three-year period. The development of the sensor system for water quality parameters represented a significant challenge for the research group, as it was to be integrated in the payload unit of the autonomous underwater vehicle in compliance with the underwater environmental conditions. The German company -4H- JENA engineering GmbH (4HJE), with experience in optical in situ-detection of nutrients, designed and built the measurement system. As a carrier platform, the remotely operated vehicle (ROV) “CWolf” from Fraunhofer-Institut für Optronik, Systemtechnik und Bildauswertung - Institutsteil Angewandte Systemtechnik (IOSB-AST) modified to an AUV was deployed. The concept presented illustrates how the measurement system can be integrated easily into the vehicle with a minimum of hard- and software technical interfaces.

## 1. Introduction

Aquaculture production increased from 5 million to 63 million tons during the last three decades, while capture fisheries production increased from 69 million to 93 million tons in the same time period [1]. Studies predict that aquaculture will supply 62% of fish destined for direct human consumption by 2030 [2]. While forming a great economic success, the ecologic impact of this increase is widely unknown. Furthermore, the industry currently lacks cost efficient observational methodology to face the challenge of determining the environmental consequences which are rooted in intensive livestock breeding with massive concentrations of fish excrements and non-controlled nutrient surpluses.

The Marine Directive of the European Union is aimed at good environmental status (GES) of the EU’s marine waters by 2020 [3]. A sustained and cost-effective monitoring of the water quality within European coastal areas is of growing importance to achieve this goal.

This paper describes an approach to improve the observational methodology of the environmental impacts by developing an automatic, unmanned underwater measuring device for sampling the water column properties. The device measures the standard physical water body properties such as temperature and salinity. Additionally, the system allows for observing other parameters such as oxygen, chlorophyll and nutrients, which are all crucial for the welfare of the naturally living resources near aquaculture sites.

Nowadays, there are many systems for monitoring fish farms. An overview about relevant works for tanks and ponds is presented in [4]. In this paper, a low-cost wireless sensor network for monitoring the water quality and the fish behavior in aquaculture tanks during the feeding process will be proposed. The system monitors the water quality parameters (temperature, conductivity, turbidity, oil layer), the tank state parameters (illumination, water level) and the fish behavior using their depth and the velocity information. The data transfer occurs via WiFi technology. The presented water quality monitoring system in [5] uses ZigBee for communication and the LabVIEW Software (National Instruments, Austin, TX, USA) for online visualization. The real-time data transmission in aquaculture monitoring systems is an important research field in recent years. Requirements like low cost and power consumption, high transmission quality, limited energy capacity, transmission rate and range have to be met. A long lasting underwater wireless sensor network using acoustic modems to solve these challenges was proposed in [6].

All presented systems above allow long-term and stationary monitoring of fish behavior and water parameters. The main issue of these systems is the spatially limited measurement in a fixed depth, where only a few measurement locations can be observed. Moreover, the equipment can stay a long time under water and can provide online data. In combination with a spatially distributed measurement approach using an underwater vehicle like an ROV or an AUV, it is feasible to combine the data and augment the stationary measured values with the vehicle measurements. Furthermore, the long-term deployment of sensors needs to consider the topic of biofouling and maritime growth that can influence the measurements. Some sensors need regular cleaning, where a short-term deployable platform such as an underwater vehicle is a good alternative.

Although several ROV applications in fish farms have been published in the past few years, the use of AUVs to solve possible tasks in and around fish farms is scarce. ROVs are in many cases a cost-effective alternative and less dangerous than using divers. In [7], an accurate navigation and guidance system for an ROV based on an ultra-short baseline (USBL) system and a Doppler Velocity Log (DVL) system was presented to support net pen inspections. This application is also the main issue in [8] where an ROV and an AUV were used. The cleaning of aquaculture nets is a further challenge for ROVs, which is presented in [9]. In [10], an ROV was used to determine the fish size for abalone fishing by using a stereo camera.

This paper includes a summary of the main results of the research project SALMON and has been previously published partly in [11,12,13]. The objectives of the project were: (i) to develop a miniaturized sensor system for real-time water quality analysis (ii) to assess the prospects for the implementation of such a sensor system in an AUV, and (iii) to examine the possibilities of using an AUV for water quality monitoring around fish farms.

## 2. Material and Methods

### 2.1. Hardware

#### 2.1.1. AUV Platform

As a carrier platform for mounting the sensor system, the Hybrid Autonomous Underwater Vehicle CWolf from the Fraunhofer IOSB-AST was used. The vehicle can operate fully autonomously or tethered with a fiber optic connection. Its modular payload concept allows for an easy sensor integration. The CWolf hardware is based on the pressure vessel construction of the widely mission tested Seawolf ROV produced by ATLAS ELEKTRONIK, Bremen, Germany.

**Figure 1:**
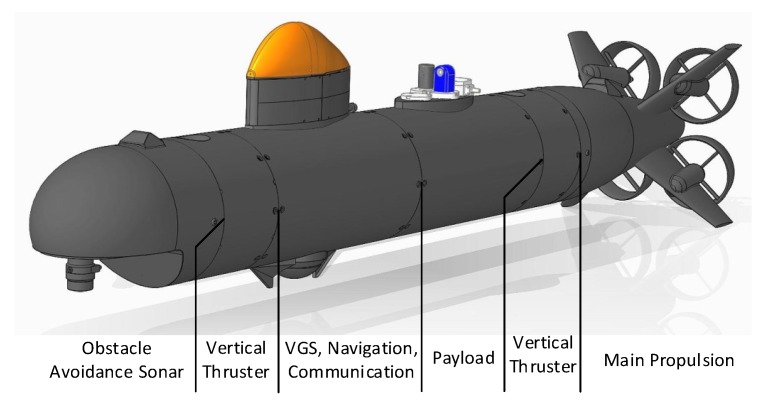
AUV CWolf.

The pressure vessel construction is segmented as shown in Figure 1. Each segment contains systems for a specific task such as propulsion, navigation, power supply, vehicle guidance or for payload sensors. With this approach, the vehicle can be reconfigured for a required application task.

The CWolf is equipped with four stern propellers for movement and maneuvering the vehicle. Different control commands of the horizontal or vertical propeller pairs result in a yaw and pitch torque. This allows the rotation of the vehicle without rudder units. Two additional vertical thrusters at the bow and stern are used for hovering and for pitch control during low speed operation. Due to the modular concept, additional horizontal thrusters can be added so that the vehicle can move sideways. This concept also allows for an easy integration of the application task with a minimum of hard- and software technical interfaces. Different kinds of payloads can be integrated into the pressure hull segment. For different payloads, different hull segments can exist and this makes the payload exchange very simple. A flange mechanically connects the segments and a bus system provides standard power supply and communication interfaces such as Ethernet, RS232 and Controller Area Network (CAN). The vehicle base platform provides accurate navigation data for vehicle guidance and geo-referencing the measured values. It consists of an inertial navigation system (INS) using several technologies such as a fiber optic gyro, a Doppler-velocity log, Global Positioning System (GPS), pressure sensors and USBL localization. The most important technical data of the AUV is shown in Table 1.

#### 2.1.2. Sensor System

The company -4H- JENA engineering GmbH (4HJE) (Jena, Germany) [14] developed and manufactured a miniaturized sensor system for the payload unit. Table 2 shows the main parameters of the system. Some of these parameters are easily measurable using standard sensors available on the market. However, the nitrate measurement is a difficult process. Through the limited space conditions in the AUV and the requirement of a low hydrodynamic resistance standard nitrate sensors like ISUS (In Situ Ultraviolet Spectrometer) [15] or SUNA (Submersible Ultraviolet Nitrate Analyzer) [16] cannot be used. These systems have a length of over 600 mm and also accuracy limitations in clouded water [17]. Although there are very accurate wet chemical methods, they are not practical for mobile underwater use. 4HJE has carried out extensive research on optical in situ-detection of nitrates in earlier projects, expertise that is used in this project. The following section presents a short introduction about the optical measurement principle and a few technical details.

**Figure 2:**
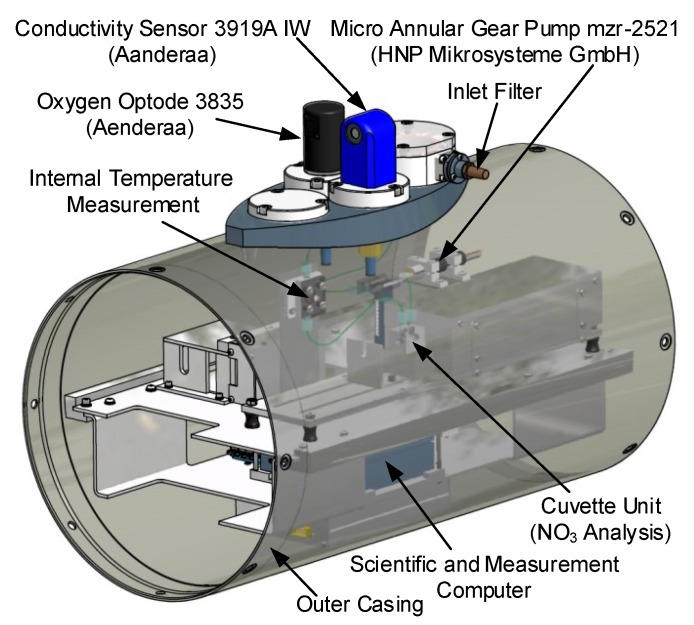
Computer-Aided Design (CAD) drawing of the sensor system (-4H- JENA engineering GmbH—4HJE).

The developed sensor system is integrated in a cylindrical hull segment matched with the diameter of the entire submerged body. Ring-shaped adapters in combination with sealing rings allow a pressure-tight connection to the rest of the submerged body. The off-the-shelf sensors used allow easy maintenance and changing from the outside without removing the segment. In addition, the inlet and outlet filters are changeable during field operations. Figure 2 shows the components and sensors of the sensor system.

The water analysis process follows the following path: Inlet Filter → Micro Annular Gear Pump → Cuvette Unit → Temperature Measurement → Outlet with Check Valve and Filter. The Scientific Computer (SC) and the Measurement Computer (MC) are situated in the lower part of the segment. These computers were connected with the internal vehicle communication unit via Ethernet. Since the nitrate measurement water had to be pumped through the vehicle, a leak sensor was installed at the bottom of the hull segment.

#### 2.1.3. Nitrate Measurement

**Figure 3:**
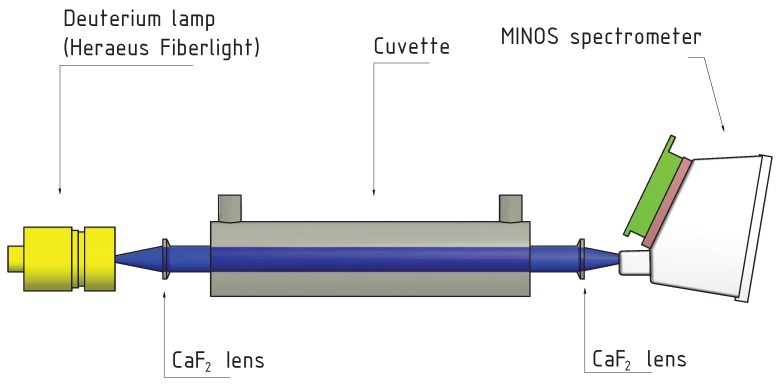
Measuring principle of nitrate determination.

**Figure 4:**
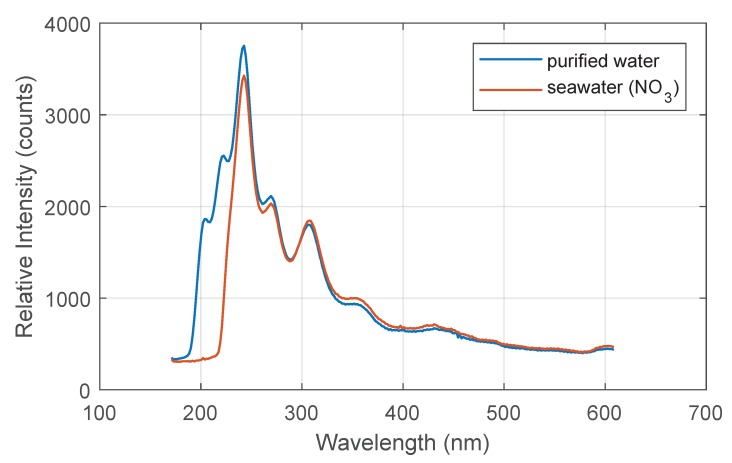
Spectra of purified water and seawater.

For the determination of nitrate, an optical method is used. The medium to be measured is pumped through a measuring cuvette via a hose system. A deuterium lamp emits ultraviolet radiation at the medium in the cuvette (see Figure 3). Some wavelengths are absorbed stronger than others by nitrate (see Figure 4).

The integrated spectrometer detects the intensity of the different wavelengths. Using stored spectra of purified water or seawater with known nitrate concentrations, the current nitrate concentration can be calculated by the MC. Additional factors influencing the measurement are the temperature of the measured medium, as well as other components in seawater. These are considered in the calculations.

**Figure 5:**
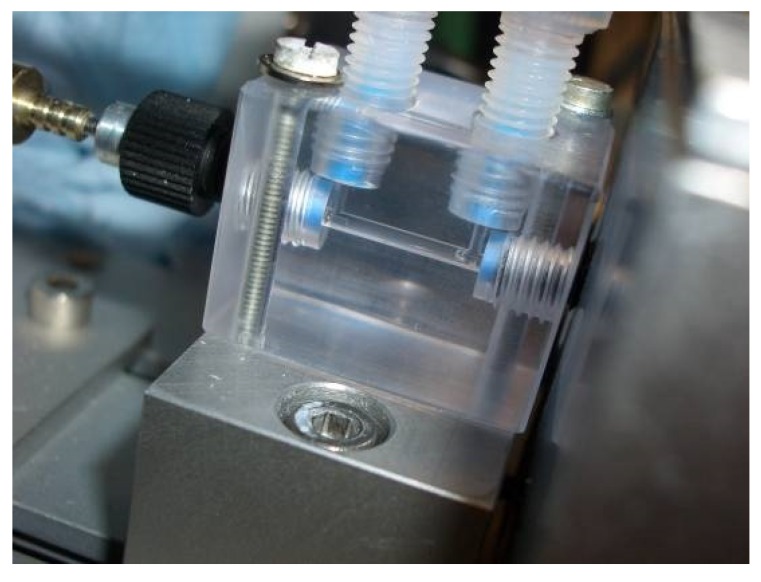
1 cm cuvette for nitrate measurement.

To adjust the measuring range of the system, the length of the cuvette must be selected accordingly. In a longer cuvette, more light is absorbed even when the nitrate concentration is low, thus the sensitivity increases with the length of the cuvette. If the cuvette is too long, too much light is absorbed and the spectrometer receives too little values for the calculation. The cuvette, which is used in the mobile analyzing unit has a length of 1 cm and is shown in Figure 5.

### 2.2. Software

The modular control software *ConSys* [18] is responsible for controlling and monitoring the base functionalities of the autonomous vehicle. This software is a development of the Fraunhofer IOSB-AST group and has already been used successfully in various underwater projects of this group.

#### 2.2.1. Communication Concept

**Figure 6:**
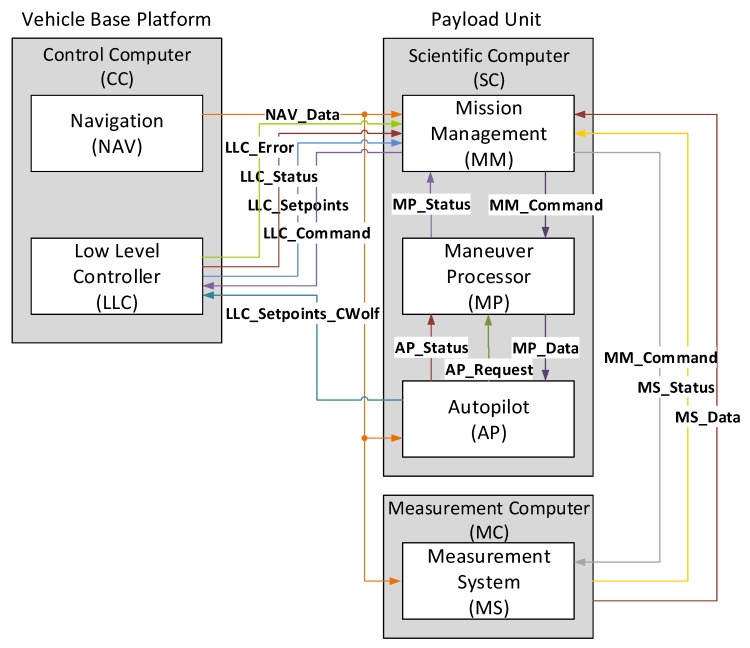
Interfaces and message telegrams between the computers.

The algorithms for navigation, control and monitoring the electrical components (power supply, computer equipment, actuators and sensors) run on the Control Computer (CC), which is located on the vehicle base platform. It provides an interface to receive navigation data, the vehicle status and error messages and controls several actuators in the Low Level Controller (LLC). The vehicle guidance runs as a backseat driver on a Scientific Computer (SC), which is located in the payload unit. A Measurement Computer (MC) for the water quality measuring management is also located there. For communication between the applications on the computers, the User Data Protocol (UDP) is used. This ensures operating system and compiler independency of the interacting software programs. Figure 6 shows the interfaces between the programs and the defined message telegrams for the exchange of information/data and for the delivery of control commands.

The telegram *LLC_Command* is needed to activate the vehicle control from the SC. Therefore, it can be switched to a *controlled mode*, a *direct mode* or *off mode*, when the SC finishes the vehicle control. In *controlled mode*, the LLC requires a heading, depth and speed command. In the SALMON project, *direct mode* was used whereby the SC controls the vehicle propulsion engines directly. It has to send a *LLC_Setpoints_CWolf* telegram, which includes the Pulse-width modulation (PWM)-values for each single motor.

The CC distributes the actual navigation data every 100 ms to all software modules. This is more than what is used by the SC and MC to handle the mission, to control the vehicle and to record the water quality measurements. The *NAV_Data* telegram includes the actual vehicle location (latitude, longitude and depth values), the vehicle orientation, speed, and height over ground values. The LLC of the CC communicates between the motor hardware and the CC-software. It receives the *LLC_Setpoint_CWolf* and *LLC_Setpoint* telegrams and sends *LLC_Status*, containing the current motor status, and *LLC_Error* telegrams, when an error has occurred. The unique identification of telegrams occurs with a header at the beginning of a telegram, which contains a message Identification (ID) and the message-payload length.

#### 2.2.2. Mission Planning

To create a mission plan, a menu-guided planning system was used. This system evolved from the planning system developed in the EU project GREX [19] and considers the experiences gathered there. The user will be presented with information and dialogs, which are necessary to solve the actual planning task step by step. The typical planning sequence includes three stages:

(1) Defining the sea chart/area of interest,
(2) Selection of the vehicle, and
(3) Build a plan using defined mission elements.

**Figure 7:**
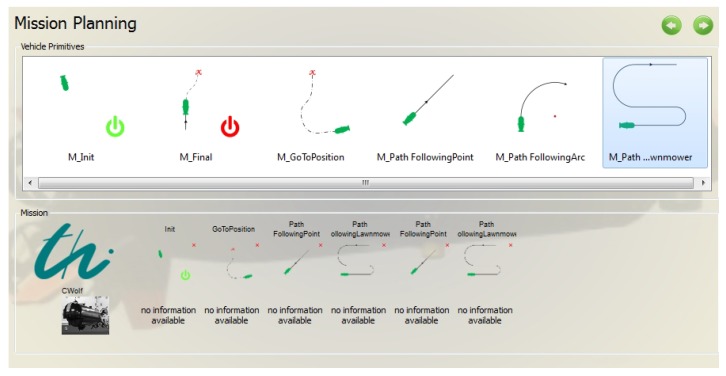
User menu for element-based planning of a mission.

Figure 7 shows the possible mission elements (vehicle primitives). Available elements include an initial and a final element (to define the start and goal position of the mission), the three base maneuver elements, (waypoint, line and arc) and the complex mission element meander. The configuration of several elements can be started by choosing the respective element in the lower half of the window. The generated mission plan will be stored in a text file. At the beginning of a mission, the Maneuver Processor loads and parses this text file in a map structure to use it during the mission.

#### 2.2.3. Dive Profiles

**Figure 8:**
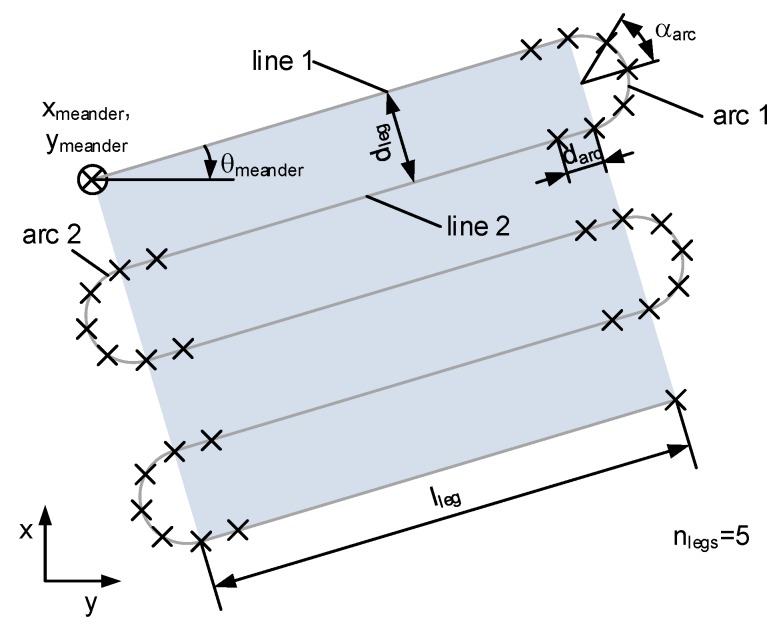
Horizontal parameters of the meander element.

The dive profiles are yo-yo style and arranged in a meander to cover the whole area. The mission element meander is specified by the following parameters: start position $x_{meander}$, $y_{meander}$, maximum depth $z_{max}$, rotation $\theta_{meander}$, leg length $l_{leg}$, distance between two legs $d_{leg}$ and the number of legs $n_{legs}$. Figure 8 shows several parameters to define a waypoint list according to the horizontal path for the meander element. The angle $\alpha_{arc}$ will be used for the reproduction of the arc element. This also defines the angle differences between adjacent waypoints on the arc. To generate a smooth transition between a line segment and an arc, a waypoint will be positioned before the start and behind the end position of the arc at a distance of $d_{arc}$. In the figure below, these “arc”-waypoints are signed with an “x” mark.

**Figure 9:**
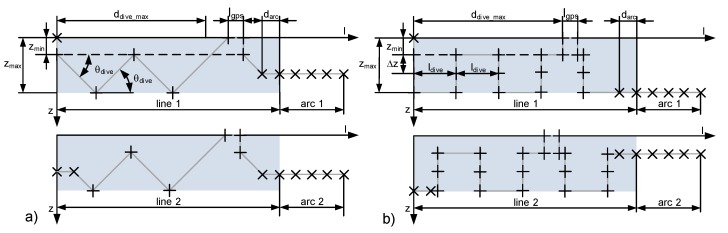
Vertical dive profiles (**a**) saw tooth profile; (**b**) square profile.

To detect the water quality parameters in several depths, two dive profiles were preferred. The first has a saw tooth shape (see Figure 9a) similar to a Slocum Glider dive profile [20]. This allows the recording of measurement data at every depth in the least amount of time using a minimal number of course changes. Therefore, the area of interest can be recorded in a minimal duration with small energy consumption by the AUV. This dive profile requires fast response times of the sensors in comparison to the changes in value of the water quality parameters during the “dive-up” and “climb-to” maneuvers. At the beginning of the mission, the AUV uses the thruster to arrive to the depth $z_{min}$. After that, the AUV uses the propellers and starts with the diagonal maneuvers. In case of complex vertical changes in the parameters or sensors with large response times, stepwise “dive-up” and “climb-to” maneuvers executed in hover mode can be used. This dive profile requires more energy and examination time for the area of interest in comparison to the saw tooth dive profile. Figure 9b shows this defined profile. To support the navigation with actual GPS positions, a surfacing is required when the distance traveled by the underwater vehicle reaches $d_{dive\_max}$.

## 3. Results

### 3.1. Test Description

**Figure 10:**
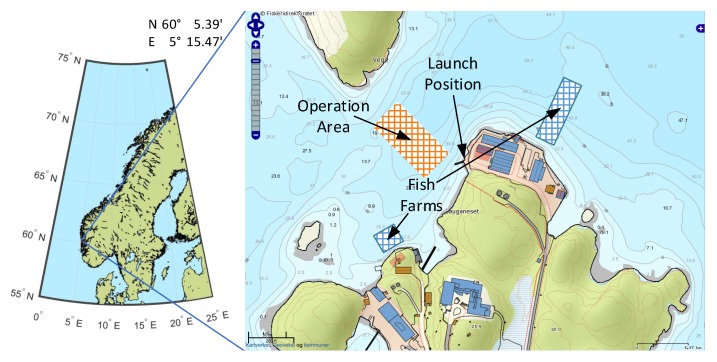
Operation area, Austevoll, Norway.

The field trials of the prototype were carried out in the second half of October 2013. The operation area is located about 30 km south of Bergen, Norway on the island of Austevoll near the experimental fish farms of the Institute of Marine Research (Figure 10). The goal of the trials was:

(i) to test the autonomous guidance of the vehicle and the sensor system in practice, and
(ii) to monitor the water quality around the fish farms.

To receive the current vehicle position and its status as well as the measurement data online, the vehicle was tethered via a fiber-optic cable also during the autonomous missions. This allowed the manual control of the vehicle in case of an emergency, like an inrush of water or a collision situation with a passing ship.

The first trial focus included the validation and adaption of the vehicle model and the controller design on the used vehicle structure as well as the tests of the guidance algorithms on the vehicle and the developed mission planning system. During the individual dives, the sensor system was active and logged water quality data. These trials occurred in a sheltered bay without shipping traffic. The access to the water occurred by a ramp using the transport trolley.

**Figure 11:**
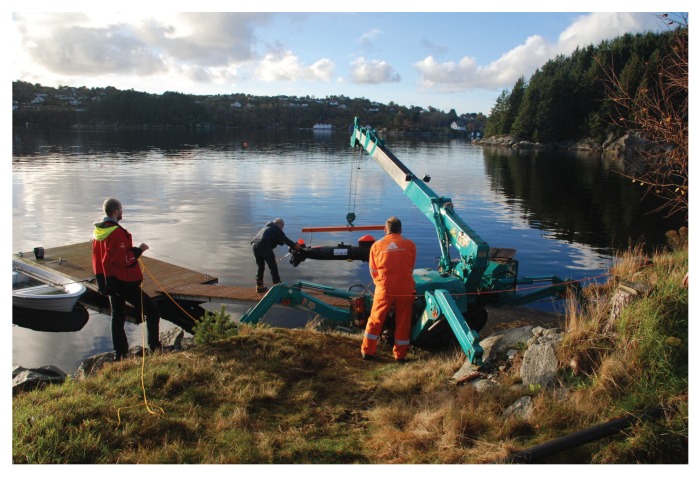
Launching of the vehicle near the fish farms in Austevoll.

The final trials were conducted between the two fish farms (see Figure 10). To launch the vehicle near the operation area, a mobile crane was used (see Figure 11). This requires careful work to avoid an impact of the sensitive sensor system or the propellers with the land stage or the transport trolley.

**Figure 12:**
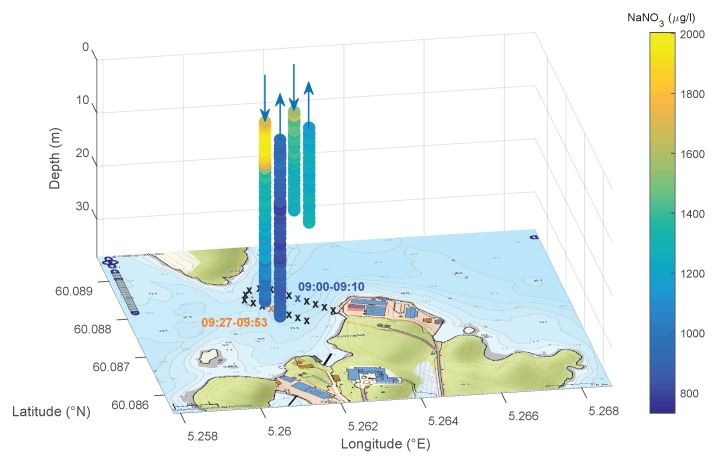
Defined mission plan and nitrate concentration for the two dive maneuvers during the mission in Austevoll on 31 October 2013.

For the mission, a meander-shaped plan consisting of 45 waypoints was defined. The farm locations were also considered in the mission planning as ‘no go area’. This allows for keeping a safe distance to the fish farms to avoid possible interactions between the AUV and the farms. The AUV headed for the individual waypoints (black marked ‘x’ in Figure 12) and dove up to the deepest depth in the bay using a square dive profile, which is presented in Section 2.2.3. The choice of this profile shape results from the limited space in the operation area. The first dive maneuver had to be interrupted, as there was a risk of a cut through the fiber optical cable by a fishing boat.

### 3.2. Analysis of the Logged Data

**Figure 13:**
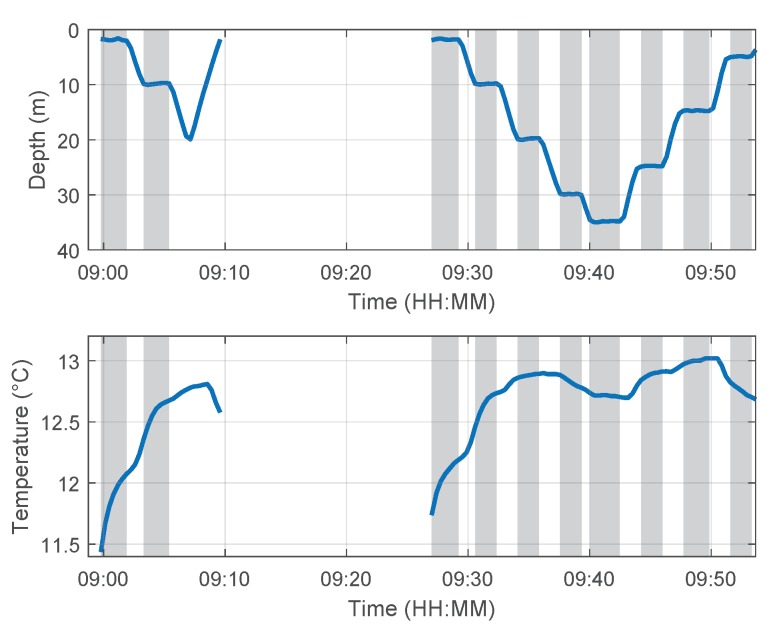
Depth and temperature data of the mission in Austevoll on 31 October 2013.

**Figure 14:**
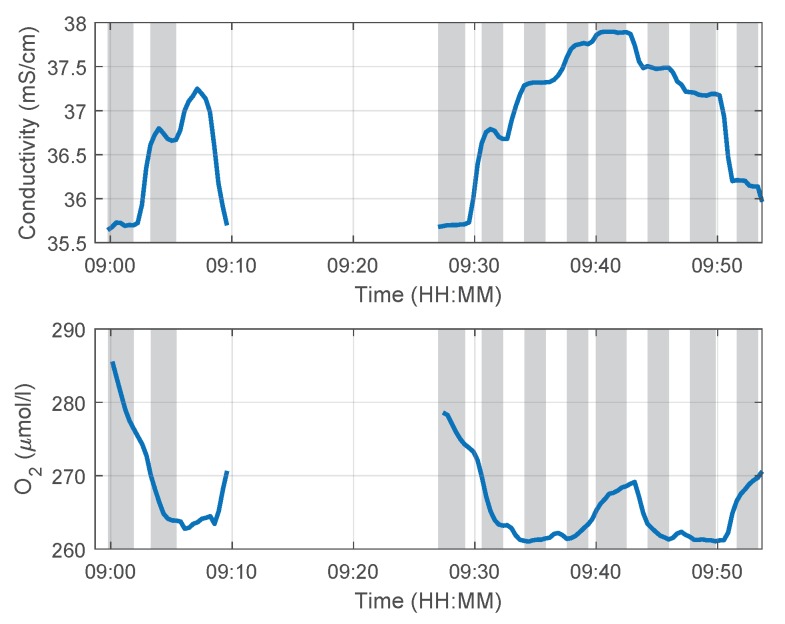
Conductivity and oxygen data of the mission in Austevoll on 31 October 2013.

Figure 13 and Figure 14 show the logged data of the depth, temperature, conductivity and oxygen for the two dive maneuvers. The gray shaded areas indicate the time ranges where the vehicle reached a new waypoint and holds a defined depth level for approximately 100 s. The vertical speed (depth rate) was 0.1 m/s. The time lag of the temperature measurement is the result of the time constant of the temperature sensor ($\tau_{Temperature}$ < 10 s [21,22]), the signal filtering and the insufficient incident flow of the sensors as a result of turbulences especially during the downward dives and hovering phases. (The time constant τ is derived from the step response of the first-order element as the duration of the time interval, required to reach 63.2% of the steady-state value of the step response [23]. The parameter settling time $T_{90}$ is often found in data sheets to describe the time characteristic of a sensor. This time is required to reach 90% of the steady-state value of the step response. In case of a first-order element and a negligible dead time in step response, the following relations to the time constant τ exist $T_{90}$ = 2.3 τ, $T_{99}$ = 4.6τ, $T_{63}$ = τ.) It can be seen in Figure 14 that the conductivity around the fish farms increases with depth. This behavior is plausible because the minerals, such as salts from the feed, settle to lower depths. The oxygen level changes in Figure 14 correlate indirectly with the temperature. The oxygen concentration decreases with increasing water temperature, which is an important aspect for aquacultural production [24].

**Figure 15:**
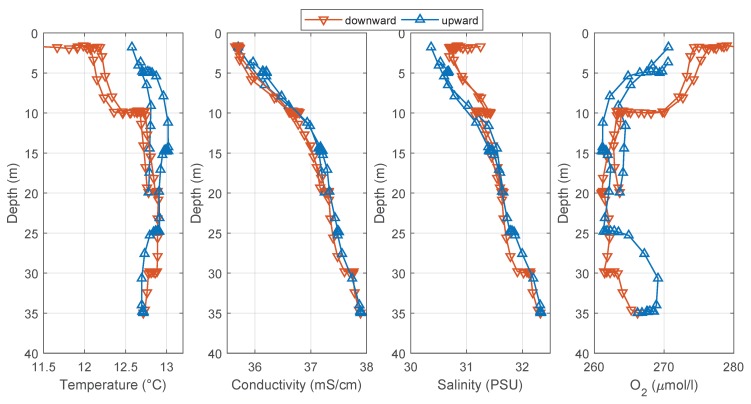
Data progressions of the mission in Austevoll on 31 October 2013.

The data profiles of the two dives are shown in Figure 15. It can been seen that there is a difference between the downward and the upward dive profiles accented by the plateaus during the defined depth levels. One reason for this behavior is the large time delay in the temperature progression, as described previously. Since the temperature information will be used in the calculation for salinity and oxygen, these plateaus can also be found there. The influence of the time delay on the conductivity measurement ($\tau_{Conductivity}<$ 1.3 s [21]) is negligible, which can be seen in the similar conductivity progressions for the downward and upward dives. The calculation of the salinity with the method described in [25] provides the same curve progression as shown in Figure 14, which is the result of the internal sensor calculation. For the salinity calculation, the function sw_salt of the SeaWater MATLAB library from CSIRO [26] was used. The input variables of this function are conductivity, temperature and pressure. The large response time of the oxygen sensor ($\tau_{Oxygen}<$ 25 s [22]) is also responsible for the plateaus in the oxygen progressions.

**Figure 16:**
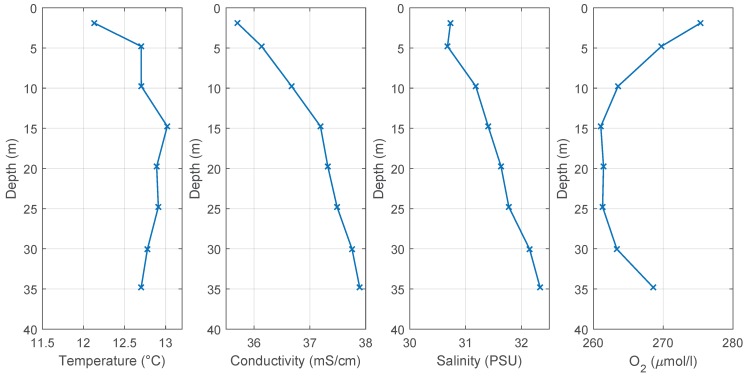
Data profiles of the mission in Austevoll on 31 October 2013.

**Figure 17:**
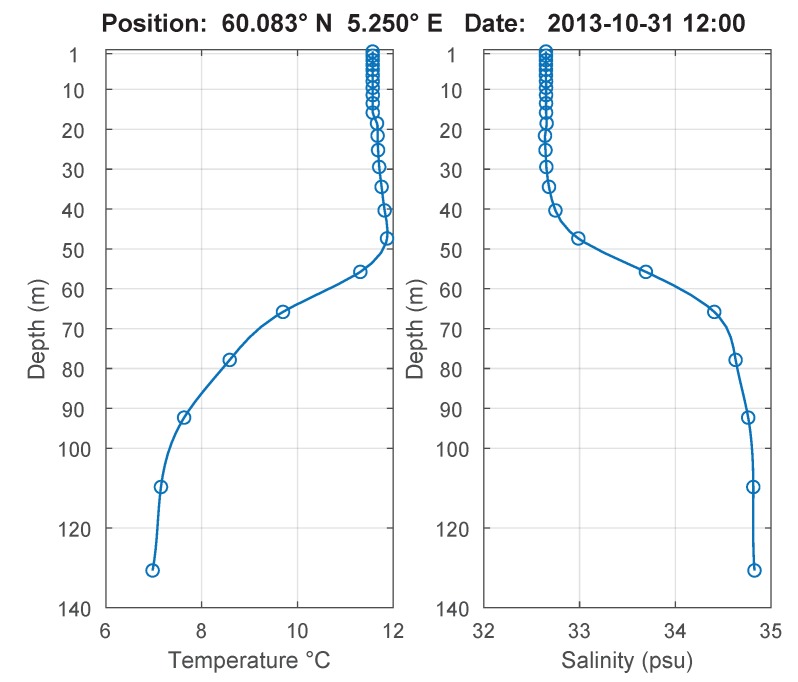
Temperature and Salinity Profile from the CMEMS model on 31 October 2013.

The generated depth profiles using the mean of the data values after the settling process in the individual depths (right sides of the gray areas in Figure 13 and Figure 14) are shown in Figure 16. Figure 17 shows the temperature and salinity profile from an ocean model of the Copernicus Marine Environment Monitoring Service (CMEMS) [27]. The principle profile shapes matched well with the logged profiles. The differences result from the inaccuracies of the model near the coast, the influence from the fish farms and the fresh water flow from local rivers.

Figure 12 shows the nitrate concentration as bars of the two dive profiles separated for the downward and upward process for a better illustration. The position and the time range of the dive maneuvers are marked with colored “x” and font on the map. The high concentration of nitrate in the surface waters is a result of the discharge of nutritive substances from the fish farms and in flow from local rivers. The analysis of the ocean current data from CMEMS in the operation area during the trials shows an offshore current (east direction) of 0.12 m/s up to 18 m depth and an onshore current (northwest direction) of 0.07 m/s in greater depths. These current conditions are a reason for the concentration distribution.

## 4. Discussion

### 4.1. Gained Experience and Possible Improvements

The trials in Norway show the possibilities and the limits of using an AUV for water quality analysis. The system allowed a fast analysis of the water quality parameters at every place around the fish farms. However, due to the heaviness of the AUV (135 kg), the launching process using the ramp or the crane was very challenging. Three employees were required for this. Here, a decrease of the vehicle-payload mass relation is desirable, which is currently 8.3 to 1. Man-portable AUVs like a REMUS 100 (Hydroid, Inc., Pocasset, MA, USA) [28], an LAUV (OceanScan, Leça da Palmeira, Portugal) [29] or an Iver3 (L3 OceanServer , Fall River, MA, USA) [30] would have the ideal weight, but they cannot implement a 15 kg payload unit. This requires more powerful drive systems and additional battery units to work under real ocean current conditions. Likewise, a hover mode with the basic versions of these vehicles is not feasible. Furthermore, the definition of the mission plan and the initialization of the sensor system (flushing the measuring section, reference measurement of the spectrum of ultrapure water) required skilled personnel.

The communication via fiber optic cable, which was used during the trials, will not be used in future real operations. Two configurations are possible: (a) an acoustic link will provide low bandwidth communication for telemetry, online measurement data transfer and emergency situations; (b) a collision avoidance system will work on board, which is described in [31]. This system uses the information from the obstacle avoidance sonar at the front of the AUV (see Figure 1) and generates setpoint commands for the autopilot controllers in a collision situation. Previous projects have shown problems for a reliable detection of fishing nets and mooring lines by the sonar.

For future use, a simplification of the sensor system is necessary. This includes an automatic initialization process and a transparent depiction of the data. It is also conceivable to relocate the water-bearing sensor parts of the nitrate measurement outside of the hull segment. This leads to a smaller and more robust system as well as to an increase in operational safety because no sample water needs to be pumped into the vehicle. In addition, the correct incident flow of the sensors with sample water in hover mode has to be analyzed in detail. The time delay of the standard sensors was noticeable. In case of a mission consisting of saw tooth profiles, an additional correction algorithm or a sensor unit which has smaller response times installed in gliders [32] have to be used. The shipping traffic around the fish farms and the related high risk of collision with the AUV was underestimated at the beginning of the trials.

### 4.2. Modular Sensor System

**Figure 18:**
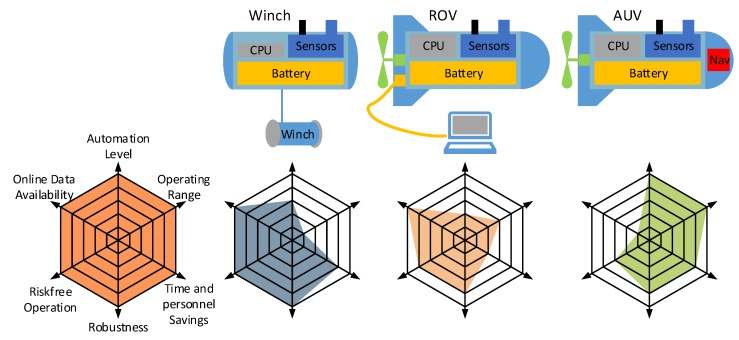
Modular sensor system with schematic and spider web diagrams for Winch, ROV and AUV.

The gained experience and the discussions with the employees of the fish farm show that an AUV is not the ultimate solution for fish farms. They need an easy-to-use system with a short lead and follow-up time. Based on these considerations, a modular sensor system will be favored (see Figure 18). This system has several extension levels with a growing degree of properties such as autonomy and operation range (see spider web diagram in Figure 18 (A spider web diagram allows the visualization of multiple qualitative variables in a two-dimensional chart. Every variable is represented by an axis. A high axis value corresponds with a good quality. By connecting several axis values with lines, a star figure is shown. The value of the resulting figure surface corresponds with the total quality.)). The base module includes the sensors, the battery and a computer unit covered in a pressure hull. The first level consists of a winch on the seabed and the sealed base module (see Figure 18 left). The sensor system can be lifted automatically or controlled by an operator. Because the park position of the sensor system is on the seabed, the likelihood of a collision or the obstruction of shipping traffic is very low. The disadvantage is the locally limited analysis of the water quality parameters in a water column. This can be done by the extension of the base module with actors and a cable-based or an acoustical communication system. This allows an expansion of the operation radius, which is restricted by the cable length or the maximum transmission range of the acoustic modem. Such a remotely operated vehicle (ROV) allows the online monitoring of the water quality parameters and the control of the vehicle during the mission. It is also possible to react to unforeseen events like ship traffic or concentration anomalies. An autonomous underwater vehicle (AUV) reaches the highest level of autonomy. This requires an exact navigation system on board and additional software and hardware modules to achieve an autonomous behavior (collision avoidance, condition monitoring). A mission plan with the defined tasks is sent to the vehicle before beginning a mission (see Section 2.2.2). Since an interaction with the vehicle is not possible during the mission, the logged data can be analyzed only after the mission. The usage of a mobile carrier platform required additional personnel and an expenditure for the preparation and follow-up of the mission. Intuitive handling in all autonomy levels is an important requirement of future use in fish farms.

## 5. Conclusions

In this paper, we present the concept, the technical implementation and the practical application of a miniaturized sensor system integrated into an autonomous underwater vehicle (AUV) for real time acquisition of water quality parameters around fish farms. We demonstrated the possibilities and the restrictions of using autonomous underwater vehicles for water quality analysis for aquaculture and coastal areas. Interested readers will find detailed information about the operating principle of the sensor system and the AUV. Furthermore, we discussed how important an intuitive and efficient handling of a modular sensor system is for future users.

## Figures and Tables

**Table 1:** Technical data of AUV CWolf.

Parameter	Value
Length	2.20 m
Diameter	0.30 m
Weight in air	135 kg
Max. speed	6 kn
Endurance at 3 kn	3 h
Payload	15 kg

**Table 2:** Parameters of the sensor system.

Parameter	Measuring Range/Value
Sodium nitrate NaNO${}_{3}$	43–2000 μg/L
Oxygen concentration O${}_{2}$	0–500 μmol/L
Conductivity σ	0–75 ms/cm
Temperature *T*	−5–40 ${}^{\circ }$C
Measurement cycle	1 s (O${}_{2}$, σ)/ 5–10 s NaNO${}_{3}$
Power Supply (Computer)	12 V
Power Supply (Sensors)	19–25 V
Energy Consumption	12 W (12 V)/10 W (22 V)

## Abbreviations

The following abbreviations are used in this manuscript:

4HJE	-4H- JENA engineering GmbH
AUV	Autonomous Underwater Vehicle
CC	Control Computer
CMEMS	Marine Environment Monitoring Service
DVL	Doppler Velocity Log
GES	Good Environmental Status
INS	Inertial Navigation System
LLC	Low Level Controller
MC	Measurement Computer
ROV	Remotely Operated Vehicle
SALMON	Sea Water Quality Monitoring and Management
SC	Scientific Computer
UDP	User Data Protocol
USBL	Ultra-Short BaseLine

References
